# Process-based screening of porous materials for vacuum swing adsorption based on 1D classical density functional theory and PC-SAFT†

**DOI:** 10.1039/d4me00127c

**Published:** 2025-01-01

**Authors:** Fabian Mayer, Benedikt Buhk, Johannes Schilling, Philipp Rehner, Joachim Gross, André Bardow

**Affiliations:** a Energy & Process Systems Engineering, Department of Mechanical and Process Engineering, ETH Zurich Zurich Switzerland abardow@ethz.ch; b Institute of Thermodynamics & Thermal Process Engineering, University of Stuttgart Stuttgart Germany

## Abstract

Adsorption-based processes are showing substantial potential for carbon capture. Due to the vast space of potential solid adsorbents and their influence on the process performance, the choice of the material is not trivial but requires systematic approaches. In particular, the material choice should be based on the performance of the resulting process. In this work, we present a method for the process-based screening of porous materials for pressure and vacuum swing adsorption. The method is based on an equilibrium process model that incorporates one-dimensional classical density functional theory (1D-DFT) and the PC-SAFT equation of state. Thereby, the presented method can efficiently screen databases of potential adsorbents and identify the best-performing materials as well as the corresponding optimized process conditions for a specific carbon capture application. We apply our method to a point-source carbon capture application at a cement plant. The results show that the process model is crucial to evaluating the performance of adsorbents instead of relying solely on material heuristics. Furthermore, we enhance our approach through multi-objective optimization and demonstrate for materials with high performance that our method is able to capture the trade-offs between two process objectives, such as specific work and purity. The presented method thus provides an efficient screening tool for adsorbents to maximize process performance.

## Abstract 2

Integrating a thermodynamic model for solid–fluid interactions into a process model to screen nanoporous materials for carbon capture.

## Graphical abstract

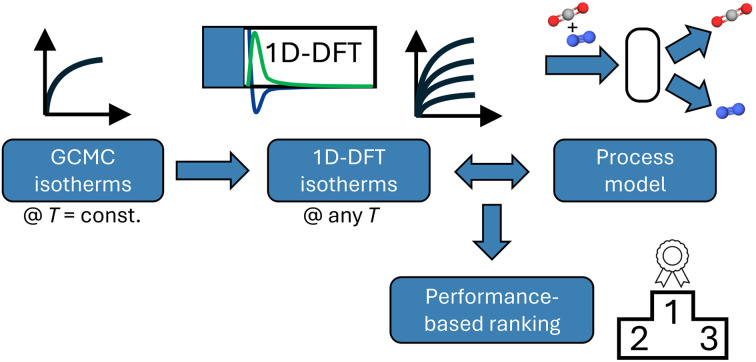

**Design, System, Application**

This study demonstrates a novel method for screening porous materials tailored for carbon capture applications by pressure and vacuum swing adsorption processes. By integrating a thermodynamic model for adsorbent properties based on classical density functional theory into a process model, our approach efficiently evaluates process performance metrics for adsorbent databases. Our method identifies optimal materials and process conditions based on process optimization and performance metrics crucial for adsorption-based carbon capture technologies. Demonstrated by a point-source carbon capture case study at a cement plant, our method significantly enhances adsorbent evaluation and selection, moving beyond simple heuristics to a rigorous performance-driven approach. This real-world application highlights the method's practical relevance and potential impact, contributing to the development of more efficient carbon capture systems. Consequently, our method addresses the pressing environmental challenge of reducing CO_2_ emissions from industrial sources and provides an efficient tool for designing carbon capture applications.

## 1 Introduction

Separation technologies are an important building block of a sustainable chemical industry.^1^ Especially, gas separation is expected to play a significant role due to the need for carbon capture,^2^ air separation,^3^ or biogas purification.^4,5^ In particular, carbon capture is a relevant technology for unavoidable CO_2_ emissions, *e.g.*, from the cement industry.^6,7^ Furthermore, carbon capture in combination with carbon storage offers the potential to generate negative emissions, *e.g.*, through capturing and storing CO_2_ from the combustion of biomass (bioenergy with carbon capture and storage, BECCS), or storing CO_2_ that is captured directly from the air (direct air capture with carbon storage, DACCS).^8,9^ Consequently, both industry and society have the need to separate CO_2_ from other gases.

So far, in industrial applications, carbon capture from flue gas is mostly based on liquid absorption methods (*e.g.*, using aqueous amines).^10–15^ A promising alternative to absorption-based separation is the separation by adsorption using solid materials, so-called adsorbents.^16–22^ However, the choice of the adsorbent for a separation process is not trivial but strongly influences the performance of an adsorption process. In the literature, many approaches are therefore presented for a systematic choice of the adsorbent, *e.g.*, by screening a given set of materials.^23–29^ Some approaches overcome the limitations of a screening by designing the adsorbent.^30–32^ However, both the screening and the design approaches often rely on material heuristics instead of a process-based objective function or surrogate models.^23^ To assess adsorbents based on the process performance, the full process cycle needs to be examined and, therefore, a process model is necessary.^26,33,34^

For a fast evaluation of many materials, the process model has to be computationally efficient. In this work, we use the equilibrium model for pressure swing adsorption (PSA) and vacuum swing adsorption (VSA) presented by Maring and Webley.^35^ The model simplifies the process and provides process performance indicators such as specific work, purity, and recovery with a reasonable computational effort.

The assessment of process performance indicators of a PSA or VSA process requires information on the interaction between the fluids and the solid adsorbent. Therefore, in addition to the process model, a thermodynamic model is also needed to describe the adsorption. Maring and Webley^35^ describe adsorption by the dual-site Langmuir isotherm model. In adsorbent screening studies, the dual-site Langmuir isotherm model is also commonly used.^23,36^ The dual-site Langmuir isotherm model is an empirical isotherm model that can be fitted to either experimental or simulated data of adsorption isotherms. Empirical isotherm models reproduce adsorption isotherms in a simplified way while retaining accuracy. However, empirical isotherm models possess a limited capability for temperature extrapolation and need to be fitted to each adsorbent–fluid combination individually.

Sauer and Gross^37^ presented a novel model to calculate adsorption properties based on one-dimensional classical density functional theory (1D-DFT) and the perturbed chain statistical associating fluid theory (PC-SAFT) equation of state. In a previous work,^38^ we showed for CH_4_, N_2_, and CO_2_ that the 1D-DFT model can accurately extrapolate in temperature and transfer parameters to other fluids and, therefore, overcomes the limitations of empirical isotherm models. The 1D-DFT model only needs to be parametrized to a single isotherm of one fluid at one temperature to calculate the isotherm data at any other temperature and for the other fluids involved in the process. Rehner *et al.*^39^ also demonstrated that the 1D-DFT model parametrized to pure-component isotherms is capable of calculating isotherms of mixtures.

Besides isotherms, enthalpies of adsorption are necessary for process models to determine the energy demand during desorption. Maring and Webley^35^ use the Clausius–Clapeyron relation to estimate enthalpies of adsorption and, for this purpose, assume that the adsorption of both components is independent of each other. While often producing accurate results, the approach of Maring and Webley^35^ simplifies the physics within the pores. In contrast, the 1D-DFT model intrinsically and consistently calculates enthalpies of adsorption.^39^ In conclusion, the 1D-DFT model can efficiently determine all solid–fluid interactions that occur in an adsorption process.

In a previous work,^40^ we used the 1D-DFT model for the screening and design of refrigerants for adsorption chillers using a process-based objective function. However, to date, the 1D-DFT model has not been applied to the screening and design of adsorbents.

In this work, we present a framework for the high-throughput screening of adsorbent databases. For this purpose, we combine the 1D-DFT model with the equilibrium process model of Maring and Webley^35^ and implement a process optimization. The presented framework is shown to determine the best adsorbent and respective process specification for a carbon capture application using a process-based objective function and consequently does not rely on performance metrics based on material heuristics.

In section 2, we explain the process model and the 1D-DFT model, and how both models interact. We combine both models for process optimization. In section 3, we apply the presented method to a carbon capture case study and discuss the results. Section 4 offers concluding remarks on the presented method.

## 2 Method

This section describes the process model (section 2.1) and metrics to assess the performance of the process (section 2.2). The thermodynamic model and its interaction with the process model for our material screening approach is introduced in section 2.3. Furthermore, we discuss how the presented models can be used for process optimization (section 2.4).

### 2.1 Equilibrium process model

We adapt the process model of Maring and Webley^35^ for PSA and VSA processes to use it in combination with the 1D-DFT model. The process model is applicable for the adsorption-based separation of a binary gas mixture. The preferably adsorbed component of the mixture is called the heavy component, the other component is called the light component, *e.g.*, for a carbon capture process, CO_2_ is usually the heavy component and N_2_ is the light component.

**Fig. 1:**
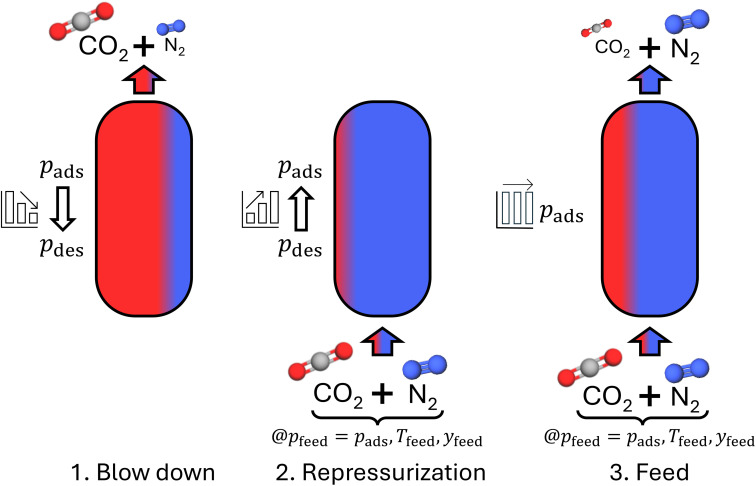
The 3 steps of the PSA/VSA process model. The colors indicate (not to scale) the concentrations of CO_2_ (red) and N_2_ (blue) in the gas phase at the end of each step.

The process aims to obtain a product stream with a high concentration of the heavy component. The process model of Maring and Webley^35^ describes the separation process in 3 steps: blow down, repressurization, and feed (Fig. 1).

The details of the process model are presented in the ESI S1.† By solving the system of equations resulting from the process model for the 3 process steps, all quantities describing the process are determined. These quantities contain the work *W*_vac_ necessary to reduce the pressure in the adsorption column, the mass *m*_product, heavy_ of the heavy component in the product stream, the amounts of substance of the heavy and the light component in the product stream (*N*_product, heavy_ and *N*_product, light_), and the amounts of substances added to the column during the repressurization and feed step (*N*_repressurization_ and *N*_feed_).

### 2.2 Process performance metrics

The performance of a material within a specific process can be quantified *via* process metrics. The equilibrium process model can calculate the specific work *w*, purity, and recovery as process metrics by1
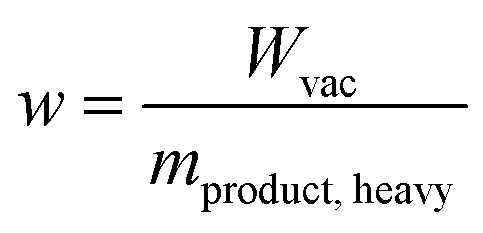
2
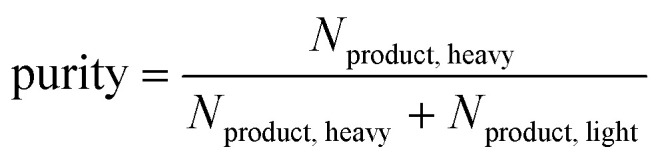
3
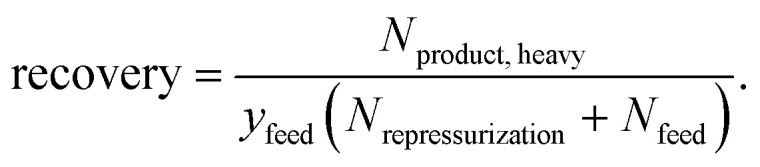
The specific work w indicates how much energy is needed for the process per mass unit of separated heavy component in the product stream (eqn (1)). The composition of the heavy component in the product stream (eqn (2)) is called purity, while the recovery indicates how much of the heavy component in the feed is captured (eqn (3)).

For large databases, an estimate of the process objectives can be beneficial before running a process simulation to quantify the potential of the materials in the database with low computational effort. The process model of Maring and Webley^35^ allows to determine an upper bound for the purity. The blow down is the only process step during which a product stream is released from the adsorber bed. The purity increases with decreasing desorption pressure because the heavy component is preferably adsorbed by the adsorbent (and consequently non-preferably desorbed). At the absolute limit, if the adsorber bed is evacuated completely, the composition of the product stream corresponds to the composition inside the adsorber bed at the beginning of the blow down, and therefore imposes an upper bound on the purity. The maximum possible purity of the process model is thus expressed by:4
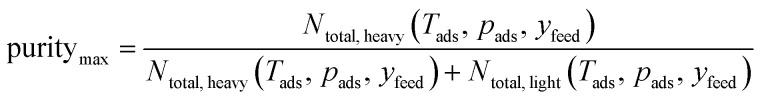
with

5 *N*_total,j_(*T*, *p*, *y*) = *q*_ads,j_(*T*, *p*, *y*)·*m*_solid_ + *ρ*_gas phase,j_(*T*, *p*, *y*)·*V*_void_, j ∈ {heavy, light}.

Materials not reaching high purities, even at vacuum conditions, can be discarded in a pre-screening before evaluating the whole process model. In real applications, more complex flow sheets (*e.g.*, reflux of waste stream) can achieve higher purities, and a theoretical maximum purity would be more complex to determine. These processes could be explored in expanded process simulations. However, the presented pre-screening approach is still expected to be useful because we assume that materials requiring additional process complexities to satisfy the purity constraint will not outperform the materials with high purity determined by the presented approach.

Due to the simplicity of the process model, the process performance metrics are expected to differ from the performance of the materials in real processes. However, the process model has been shown to still provide insights into the relative performance of different materials.^35^ Furthermore, our study's aim is to show how the 1D-DFT model can be integrated into a process model and to provide a proof-of-concept of an integrated adsorbent screening based on a physical sound adsorption model describing the solid–fluid interactions.

### 2.3 Thermodynamic model

The process model presented in section 2.1 requires uptakes and adsorption enthalpies at certain pressures, temperatures, and compositions. For this purpose, we apply the 1D-DFT model presented by Sauer and Gross.^37^ The 1D-DFT model is used through the open-source Python package FeO_s_.^39^

In the 1D-DFT model, the density distribution of the molecules within the pores of the adsorbent is calculated based on the interactions between the solid atoms and the fluid molecules. The geometries of the pores are simplified to 1-dimensional geometries, *e.g.*, slit pores, cylindrical pores, or spherical pores. For the calculations of the 1D-DFT model, the adsorbent is described by the parameters pore size *r*_pore_ (radius of spherical and cylindrical pores, pore width of slit pores), segment diameter *σ*_ss_, dispersion energy parameter *ε*_ss_, and density *ρ*_s_.

By using the approach of our previous work,^38^ 1D-DFT parameters describing the adsorbent can be determined by fitting the model to either experimental or simulated isotherms. In our previous work,^38^ we showed that 1D-DFT parameters fitted at one temperature can be extrapolated to other temperatures. Furthermore, 1D-DFT parameters can be transferred to other fluids. Due to the transferability of the 1D-DFT parameters to other fluids, we assume that the 1D-DFT parameters can also be transferred to isotherms of mixtures at any composition.^39^ For mixtures, a binary interaction parameter (*k*_ij_) is introduced in the PC-SAFT equation of state to account for non-idealities of the fluid/fluid interactions.^41^

The original process model of Maring and Webley^35^ uses the Clausius–Clapeyron relation to estimate the enthalpy of adsorption. Since the enthalpy of adsorption can be directly calculated by the 1D-DFT model, we replace the estimation based on Clausius–Clapeyron. Furthermore, in the work of Maring and Webley,^35^ the amount of substances in the gas phase within the adsorber bed is calculated with the ideal gas law. We replace this simplification by calculating the amount of substances with PC-SAFT.

### 2.4 Material screening and process optimization based on 1D-DFT model

In order to identify the best-performing material out of a large variety of materials, we automatically evaluate the process metrics for large databases of 1D-DFT parameters. However, each material requires its own process settings, here the desorption pressure *p*_des_. Therefore, a process optimization identifies the most suitable desorption pressure *p*_des_ for each material.

The optimization problem minimizes the specific work *w* as an indicator of the operational cost of the capture process. Additionally, the optimization problem constrains the purity because the sink for the product stream usually requires a high purity.^42^ All materials with a maximum possible purity below the purity constraint can be discarded because these materials cannot fulfill the constraint. For each remaining material of a database, our method then optimizes the process. Thereby, a ranking of the materials is obtained, including their optimal desorption pressure *p*_des_. The optimization problem for one material is expressed mathematically asP1
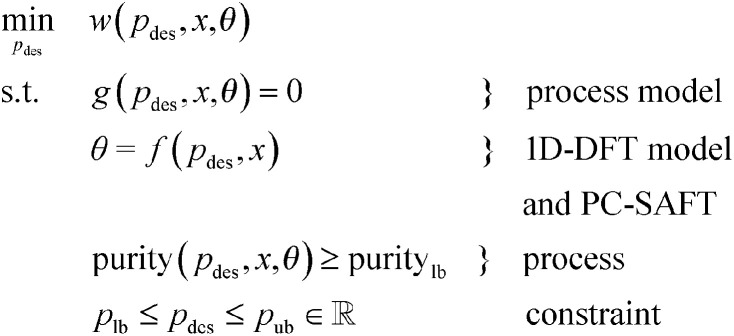
where the vector *x* denotes the process inputs, *e.g.*, temperature, pressure, and composition of the feed, as well as the 1D-DFT parameters. The fluid properties and adsorption properties (*e.g.*, enthalpies, uptakes) are described by *θ*. *g*(*p*_des_, *x*, *θ*) is the set of equations of the process model (*e.g.*, component and energy balances, see section 2.1) and *f*(*p*_des_, *x*) the set of equations of the thermodynamic model (here 1D-DFT model and PC-SAFT). The abbreviations lb and ub denote the lower and upper bounds of the pressure *p*_des_. We apply the non-linear solver Knitro^43^ to solve optimization problem P1 for each material.

## 3 Screening MOFs for post-combustion carbon capture

In this section, we apply the method presented in section 2 to a point source post-combustion CO_2_-capture case study and screen a database of metal–organic frameworks (MOFs). Section 3.1 provides more details on the case study and the used database. In section 3.2.1, we perform a pre-screening followed by a full screening based on process optimization (section 3.2.2). Finally, we analyze the results in more detail by performing multi-objective optimization (section 3.2.3).

### 3.1 Case study

Our case study considers point source post-combustion CO_2_-capture at a cement plant. Numeric values for the case study are taken from Charalambous *et al.*,^44^ so the flue gas has a temperature of 110 °C and a pressure of 1.013 bar. We take the composition values of a dry flue gas consisting only of CO_2_ and N_2_ from Charalambous *et al.*,^44^ since the process model is restricted to the separation of 2 components. The composition of the flue gas, and hence of the feed of the adsorption process, is 19.78% CO_2_ and 80.22% N_2_. Temperature, pressure, and composition of the flue gas specify the feed stream of the process since it is assumed that the capture process is installed directly downstream of the cement plant. For these specifications, a VSA process is used since the feed pressure is close to ambient pressure, so the desorption pressure has to be below ambient pressure. Thus, a vacuum pump is necessary. In accordance with the original model of Maring and Webley,^35^ all process calculations are in relation to an adsorbent mass *m*_solid_ of 1 kg, and the void fraction of the adsorber bed *ε*_bed_ is 0.37. For the ratios of heat capacities, we use the values *κ*_heavy_ = 1.28 for CO_2_ and *κ*_light_ = 1.4 for N_2_.^45^ The values of all parameters defining the case study are listed in Table 1.

**Table 1:** Overview of case study parameters for carbon capture at a cement plant

Parameter	Symbol	Value	Ref.
Temperature feed	*T*_feed_	110 °C	44
Pressure feed	*p*_feed_	1.013 bar	44
Mole fraction CO_2_	*y*_feed, heavy_	19.78%	44
Mole fraction N_2_	*y*_feed, light_	80.22%	44
Mass adsorbent	*m*_solid_	1 kg	35
Void fraction adsorber bed	*ε*_bed_	0.37	35
Ratio of heat capacity CO_2_	*κ*_heavy_	1.28	45
Ratio of heat capacity N_2_	*κ*_light_	1.4	45

### 3.2 High-throughput screening

#### 3.2.1 Pre-screening

In the first step, we parameterize the 1D-DFT parameters to a database of 775 MOFs.^44,46^ For each MOF, the database contains isotherms of CO_2_ and N_2_ at 298.15 K generated by GCMC simulations. The MOFs feature a wide range of metals, linkers, ligands, pore sizes, and topologies and are named according to the Cambridge Structural Database.^47^ The database also contains the heat capacities *c*_p, solid_ and densities *ρ*_solid_ for each material. These material properties are calculated with the software tool Zeo++.^46,48^ For the generation of the set of 1D-DFT parameters for each MOF, we use the parametrization approach presented in our previous work^38^ by fitting the model to the isotherm data generated by the GCMC isotherms.

The 1D-DFT model is fitted simultaneously to CO_2_ and N_2_ to increase the accuracy for mixtures of the two fluids. We assume spherical pores for all MOFs. CO_2_ and N_2_ are modelled by the PC-SAFT parameters provided by Gross and Sadowski.^49^ A binary interaction parameter *k*_ij_ is used to model the mixture of CO_2_ and N_2_ more precisely. The binary interaction parameter of *k*_ij_ = −0.012 is obtained by fitting vapor–liquid equilibrium calculations from PC-SAFT for the mixture of CO_2_ and N_2_ to experimental data provided by Westman *et al.*^50^

**Fig. 2:**
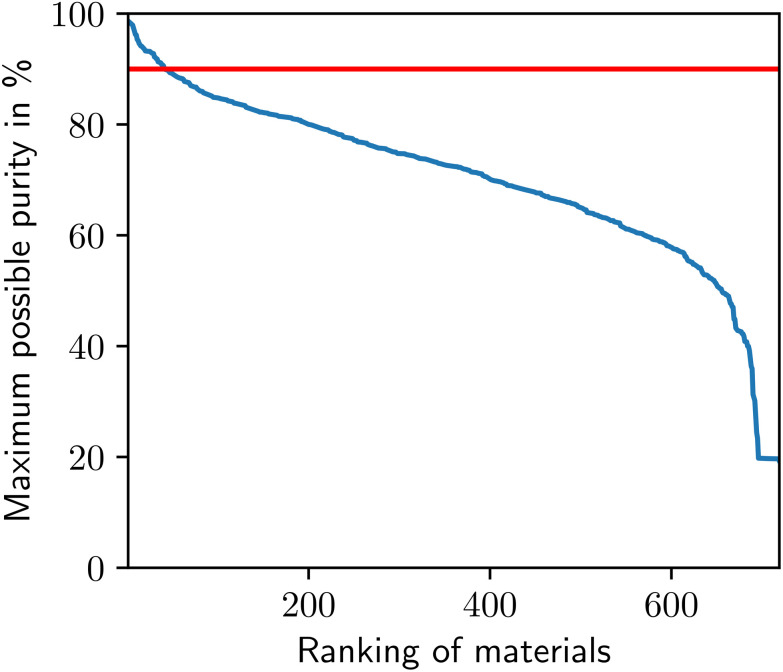
Maximum possible purity of all MOFs of the database. The red line indicates the minimum purity requirement of 90%.

As a pre-screening, we evaluate the maximum possible purity with the approach presented in section 2.2 (Fig. 2). The MOF BUSQIQ achieves the highest purity with 98.6%. 5% of the MOFs of the database can achieve high purities of 90%. Below this threshold, purities steadily drop to 60% followed by a steep drop to purities corresponding to the concentration of the feed mixture, indicating the few MOFs with very limited adsorption of CO_2_ or even favorable adsorption of N_2_. Fig. 2 shows that many MOFs cannot achieve high purities and, hence, are not relevant for post-combustion CO_2_-capture at a cement plant. To save computational costs, we do not further consider the MOFs that cannot reach purities above 90%.

#### 3.2.2 Process optimization-based screening

After the pre-screening, 41 MOFs with a maximum possible purity greater than 90% remain as candidate structures and are used in a screening based on process optimization. For these 41 MOFs, the specific work is minimized in the process optimization. A minimum constraint for the actual purity achieved in the process is set at 90%. Thereby, the desorption pressure minimizes the specific work while still maintaining a high purity. A ranking of the MOFs is obtained. The results of the 10 best-performing materials are shown in Table 2. The lowest specific work of 393 kJ kg^−1^ achieved in the screening is slightly above double the thermodynamic minimum energy requirement of 182 kJ kg^−1^ for this separation task. The best materials reach the range of energy demands reported for absorption-based carbon capture processes by Renfrew *et al.*^51^

**Table 2:** Top 10 MOFs with lowest specific work at a purity of 90%

Rank	Name	Specific work *w* in kJ kg^−1^	Desorption pressure *p*_des_ in kPa	Maximum possible purity in %
1	BUSQIQ	393	2.1	98.6
2	NAXLII	393	2.1	94.1
3	ELUJEC	394	1.6	91.9
4	WOWMEC	394	1.8	93.2
5	SISFEH	399	1.7	92.1
6	POKGEC	404	1.8	93.9
7	DOJXIK	408	1.6	93.2
8	CAKXUJ	413	1.5	96.1
9	ATUYAR	415	1.2	91.3
10	CAKXET	420	1.5	95.3

**Fig. 3:**
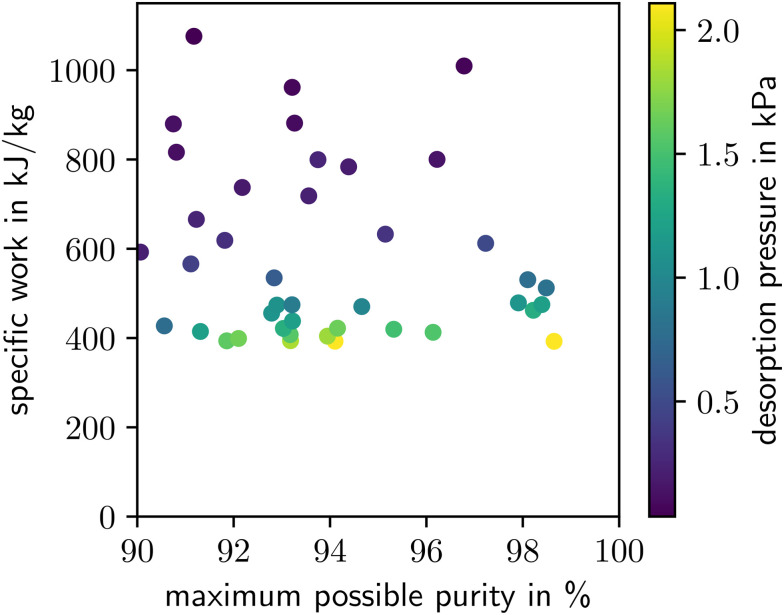
Minimal specific work and respective maximum possible purity of MOFs capable of reaching purities above 90%. In the optimization, the actual purity achieved in the process is constrained to be above 90%. The colors indicate the desorption pressure which leads to the minimal specific work.

In Fig. 3, the specific work of the process optimizations is shown in comparison to the maximum possible purity of each MOF. The color code of Fig. 3 indicates that the specific work decreases with an increase in the desorption pressure. High desorption pressures are favorable since less vacuum work is required. For all MOFs analyzed here, the actual purity of the optimal process configuration is exactly 90% because both the specific work and the purity decrease with increasing desorption pressure, and, thus, the optimal solution with minimum specific work corresponds to an active purity-constraint.

The pressure levels are lower than reported in the literature for carbon capture by VSA.^52^ However, the simplified process model requires low pressures to reach high purities, as also observed in the work of Maring and Webley.^35^ More complex process models would be necessary to evaluate the process with pressure levels closer to real applications. Still, we believe that the simplified model is valuable for efficient material screening.

The *x*-axis in Fig. 3 displays the maximum possible purity determined in the pre-screening (see section 3.2.1). The lowest specific work of 393 kJ kg^−1^ is achieved by the MOF BUSQIQ, which already achieved the highest maximum possible purity in the pre-screening. However, the maximum possible purity is a poor proxy for the process performance since the next-best MOF regarding work (NAXLII) is only on rank 16 regarding maximum purity. Materials with a comparatively low maximum purity in the pre-screening are still able to achieve good results in the process optimization-based screening. In conclusion, Fig. 3 does not imply a relation between the specific work and the maximum possible purity.

The maximum possible purity can be seen as a material-based objective since it directly depends on the selectivity of the material, while the specific work is a process-based objective. A high maximum possible purity indicates strong bindings for CO_2_ but weak bindings for N_2_. However, this relation in binding strengths does not provide information on the actual process performance because a strong binding for CO_2_ also implies an increased energy demand to desorb the CO_2_. Accordingly, Fig. 3 illustrates that a material-based objective is not sufficient to determine the performance of a material in the capture process, confirming the need for process-based objectives as demonstrated in previous studies on process design for adsorption-based separation.^23–25,53^ A material-based objective is useful in a pre-screening if, thereby, a bound on a process-based metric is imposed as shown for the purity in section 3.2.1. Gopinath *et al.*^54^ also showed that such pre-screenings to exclude infeasible regions improve the computational efficiency of molecular design. However, a process model is necessary to determine process-based objectives for the performance analysis of a material within a certain process. The necessity of a process model shows the importance of our approach to integrate the whole process into the material selection process and not solely rely on metrics based on material properties.

The results of our screening method depend on the accuracy of the 1D-DFT model, especially if a parameter set can represent both CO_2_- and N_2_-isotherms for the same material. In the ESI S2,† the CO_2_- and N_2_-isotherms calculated by the 1D-DFT model are shown in comparison to GCMC data for the 3 best-performing MOFs (BUSQIQ, NAXLII, ELUJEC). CO_2_-isotherms are underestimated and N_2_-isotherms are overestimated by the 1D-DFT model, which leads to a systematic underestimation of the process performance in our proposed method. In future work, the simultaneous parametrization of the 1D-DFT model to CO_2_- and N_2_-isotherms needs to be refined for some materials to achieve results closer to real processes.

#### 3.2.3 Multicriterial analysis of top-performing MOFs

**Fig. 4:**
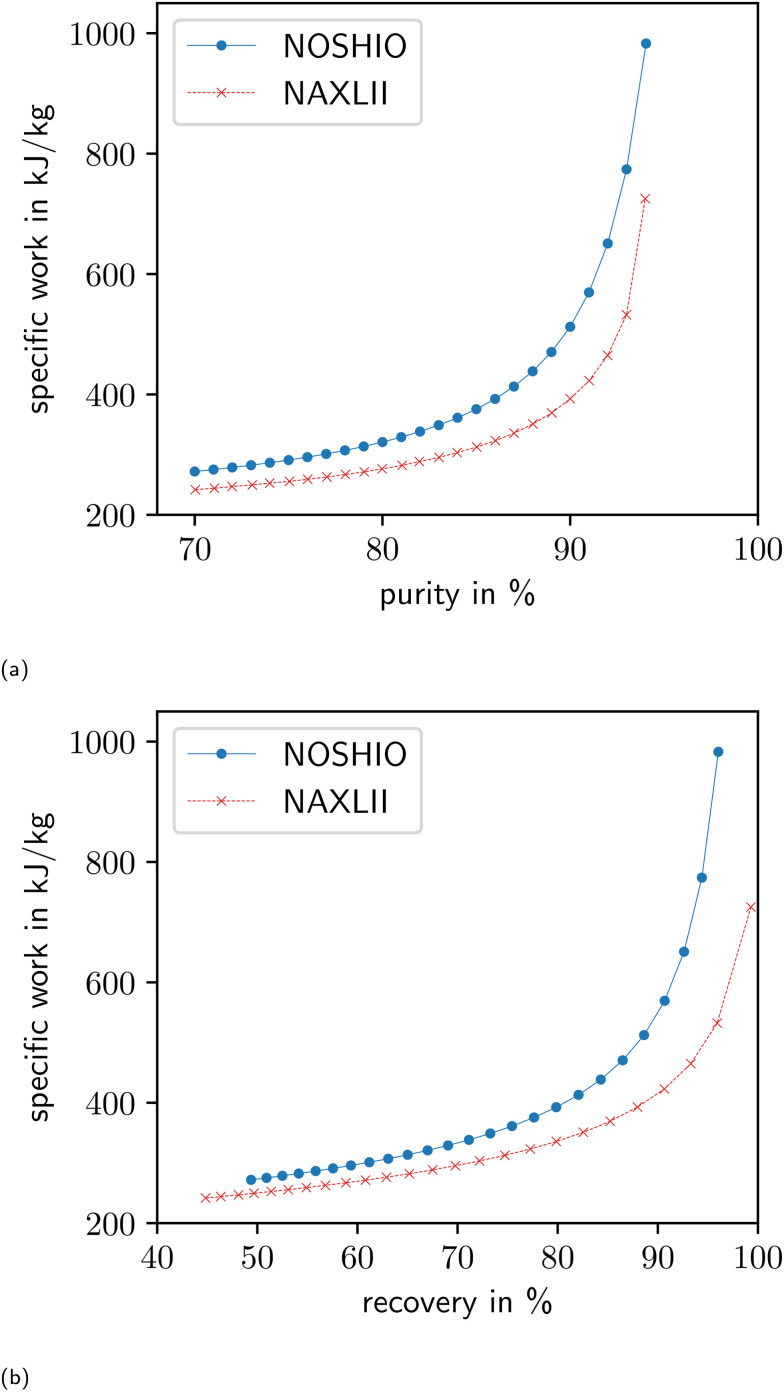
Pareto fronts for the trade-off between specific work and purity (a) as well as respective recoveries (b) for the MOFs NAXLII and NOSHIO.

Our study sets the purity-constraint of 90% arbitrarily. For this reason, the trade-offs between specific work and purity or specific work and recovery are not sufficiently captured in the material screening presented in section 3.2.2. To capture these trade-offs, we perform multi-objective optimization for the two MOF-structures NAXLII and NOSHIO. As presented in Table 2, NAXLII is the MOF with the second-lowest specific work. NOSHIO reaches the second-highest maximum possible purity of 98.5%, which is very close to the highest observed possible purity of BUSQIQ. However, with a specific work of 512 kJ kg^−1^, NOSHIO only reaches rank 22 in the screening of section 3.2.2. For multi-objective optimization, we use the *ε*-constraint method.^55^ In this approach, we minimize the specific work for different values of the purity-constraint. Thereby, the Pareto fronts shown in Fig. 4a are obtained.

NAXLII outperforms NOSHIO over the whole range of feasible purities. With an increase in purity, the slopes of the Pareto fronts increase. The increasing slopes indicate that the higher the purity, the more additional energy is required to increase the purity further.

In Fig. 4b, the recoveries of the respective optimized processes are shown. Higher purities also lead to higher recoveries. The slope of the recovery curve is lower than the slope of the purity curve, which indicates that an increase in the recovery requires less additional energy than a similar increase in purity.

The better performance of NAXLII on a wide range of purities, compared to NOSHIO, emphasizes that an assessment based on process performance is necessary and material-based heuristics are not sufficient: even though the maximum possible purity of NOSHIO is high, the specific work cannot reach the levels of NAXLII. Furthermore, the lack of feasible solutions close to the maximum possible purity of NOSHIO shows that the assessment based on the process model is necessary. The maximum possible purity solely indicates a theoretical upper bound on the purity that potentially can be reached by a material. However, the maximum possible purity does not indicate the actual performance of an adsorbent within the adsorption–desorption cycle.

## 4 Conclusions

Adsorbent selection is of major importance for the design of adsorption-based separation processes since the choice of the solid material strongly affects the process performance and, consequently, energy requirements and costs. In this work, we present a method to screen materials for pressure and vacuum swing adsorption processes based on 1D-DFT. By using a process model for the PSA/VSA process, the performance of adsorbent materials is determined within the process. Our presented method calculates adsorption properties, *e.g.*, uptakes or enthalpies of adsorption, by the 1D-DFT model, which is a physics-based and computationally efficient model. Based on a single isotherm, the 1D-DFT model determines all necessary adsorption properties. Fluid properties are determined by the PC-SAFT equation of state.

We apply our method to a point-source carbon capture case study of a cement plant. By performing a pre-screening of 775 MOFs based on the maximum possible purity within the process, we choose 6% of the MOFs that can reach purities above 90%. Next, we screen these MOFs by determining their performance within the process model. The screening is based on a process optimization that identifies the desorption pressure leading to the lowest specific work while still maintaining a constraint of 90% on the purity. Our screening approach of MOFs for carbon capture application using 1D-DFT successfully identifies the MOF with the lowest specific work necessary for the process. Thereby, we demonstrate that the 1D-DFT model is a promising tool for the design of adsorption-based processes since the description of solid–fluid interactions can be integrated into a process model. Furthermore, we show that our method is able to capture trade-offs between specific work and purity or recovery by applying multi-objective optimization. However, the results of this work are influenced by the choice of the process model. We chose a simplified equilibrium process model to demonstrate the applicability of our proposed method. In future work, if a prediction model for transport coefficients becomes available, rigorous process models can be used to achieve results closer to real-world applications. The results are also influenced by the simultaneous parametrization of the 1D-DFT parameters to CO_2_- and N_2_-isotherms, which needs to be refined in future work for some materials.

To summarize, we present a promising method to rapidly evaluate the process performance of a large number of adsorbent materials for carbon capture. The method is not limited to carbon capture and can also be applied to other adsorption-based separation processes, *e.g.*, biogas purification or air separation. Furthermore, the method can be extended to more comprehensive objective functions, *e.g.*, cost or environmental impacts.^44^ Thereby, our presented integrated screening approach can assist in choosing the optimal adsorbent for novel processes in a sustainable chemical industry.

## Data availability

The Python code developed in this work and csv-files with the parameters and results are available in the following Gitlab repository: https://gitlab.ethz.ch/epse/molecular-design-public/paper-p_vsa-process-screening/.

## Author contributions

Fabian Mayer: conceptualization, methodology, software, validation, visualization, investigation, writing – original draft. Benedikt Buhk: software, writing – review & editing. Johannes Schilling: conceptualization, methodology, writing – review & editing. Philipp Rehner: conceptualization, methodology, supervision, writing – review & editing. Joachim Gross: conceptualization, writing – review & editing. André Bardow: conceptualization, resources, writing – review & editing, supervision, funding acquisition.

## Conflicts of interest

There are no conflicts to declare.

## Supplementary Material

ME-010-D4ME00127C-s001
