# Supplementary material for: Process-based screening of porous materials for vacuum swing adsorption based on 1D classical density functional theory and PC-SAFT
Source: Mol Syst Des Eng. 2025 Jan 1;10(3):219–27. doi: 10.1039/d4me00127c (PMC11701972; doi:10.1039/d4me00127c)
Supplement: ME-010-D4ME00127C-s001 [file ME-010-D4ME00127C-s001.pdf]

Supplementary Information (SI) for Molecular Systems Design & Engineering.
This journal is © The Royal Society of Chemistry 2024

Supporting Information: Process-based screening of porous materials for vacuum swing adsorption based on 1D classical density functional theory and PC-SAFT

Fabian Mayer,† Benedikt Buhk,† Johannes Schilling,† Philipp Rehner,† Joachim Gross,‡ and André Bardow*,†

†*Energy & Process Systems Engineering, Department of Mechanical and Process Engineering, ETH Zurich, 8092 Zurich, Switzerland*

‡*Institute of Thermodynamics & Thermal Process Engineering, University of Stuttgart, 70569 Stuttgart, Germany*

E-mail: abardow@ethz.ch

S1 Process model

In our work, we use the process model of Maring and Webley [1] for PSA and VSA processes, which consists of the 3 steps blow down, repressurization, and feed. For each step, the model consists of balances for the heavy component (Equation (S1)), the light component (Equation (S2)) as well as energy (Equation (S3)). The process is adiabatic, so the temperature changes within the adsorption column during the blow down and the repressurization steps. However, the change in temperature T is not known a-priori. Furthermore, the amount of

substance ΔN entering or leaving the adsorption column and the composition y of the gas phase inside the adsorption column are not known a-priori. These 3 equations (component balance heavy, component balance light, energy balance) determine the 3 unknown quantities (temperature T, change in total amount of substance ΔN, composition y of the gas phase).

Blow down and repressurization change the pressure, and thus, the uptake of the adsorbent and the enthalpy of adsorption change continuously throughout the process steps. To address this continuous change, the blow down step and the repressurization step are discretized in small increments of the pressure change. The resulting set of equations is solved sequentially for each increment i, and the solution is used as input for the next increment. The set of equations is solved with the least-squares optimizer of SciPy.[2] Summing up the results of all increments quantifies the amount of substance of each component entering or leaving the adsorption column and the energy necessary to run the process.

$$\begin{aligned} 0 = m_{\text{solid}} \cdot \big(q_{\text{ads,heavy}}(p_i, T_i, y_i) - q_{\text{ads,heavy}}(p_{i-1}, T_{i-1}, y_{i-1})\big) \\ + V_{\text{void}} \cdot \big(\rho_{\text{gas,heavy}}(p_i, T_i, y_i) - \rho_{\text{gas,heavy}}(p_{i-1}, T_{i-1}, y_{i-1})\big) - y_{\Delta N_i} \Delta N_i \end{aligned} \tag{S1}$$

$$\begin{aligned} 0 = m_{\text{solid}} \cdot \big(q_{\text{ads,light}}(p_i, T_i, y_i) - q_{\text{ads,light}}(p_{i-1}, T_{i-1}, y_{i-1})\big) \\ + V_{\text{void}} \cdot \big(\rho_{\text{gas,light}}(p_i, T_i, y_i) - \rho_{\text{gas,light}}(p_{i-1}, T_{i-1}, y_{i-1})\big) - (1 - y_{\Delta N_i}) \Delta N_i \end{aligned} \tag{S2}$$

$$\begin{aligned} 0 = h_{\text{ads,heavy}}(\overline{p}_i, \overline{T}_i, \overline{y}_i) \cdot m_{\text{solid}} \cdot \big(q_{\text{ads,heavy}}(p_i, T_i, y_i) - q_{\text{ads,heavy}}(p_{i-1}, T_{i-1}, y_{i-1})\big) \\ + h_{\text{ads,light}}(\overline{p}_i, \overline{T}_i, 1 - \overline{y}_i) \cdot m_{\text{solid}} \cdot \big(q_{\text{ads,light}}(p_i, T_i, y_i) - q_{\text{ads,light}}(p_{i-1}, T_{i-1}, y_{i-1})\big) \\ - m_{\text{solid}} c_{\text{p,solid}} (T_i - T_{i-1}) \end{aligned} \tag{S3}$$

with increment-averaged quantities $(\overline{p}_i, \overline{T}_i, \overline{y}_i)$ defined as

$$\overline{x}_i = \frac{1}{2}(x_i + x_{i-1}) \tag{S4}$$

and

$$
\begin{aligned}
y_{\Delta N_i} &= \frac{1}{2}(y_i + y_{i-1}) && \text{for blow down} \\
y_{\Delta N_i} &= y_{\text{feed}} && \text{for repressurization}
\end{aligned}
\tag{S5}
$$

and

$$
V_{\text{void}} = \frac{\varepsilon_{\text{bed}}}{1-\varepsilon_{\text{bed}}} \frac{m_{\text{solid}}}{\rho_{\text{solid}}}. \tag{S6}
$$

The density of the gas phase $\rho_{\text{gas}}(p,T,y)$ is calculated by the PC-SAFT equation of state[3]. The 1D-DFT model provides the uptake $q_{\text{ads}}(p,T,y)$ and the enthalpy of adsorption $h_{\text{ads}}(p,T,y)$. In Equations (S1), (S2), and (S3), m_{solid} is the mass of the adsorbent. The void volume V_{void} describes the volume accessible by the gas phase and can be calculated by Equation (S6) from the void fraction ε_{bed} of the adsorber bed due to the packing of the adsorbent material (e.g., randomly packed spherical beads) and the density ρ_{solid} of the material. By solving this set of equations, the amount of substance ΔN_i entering or leaving the adsorption column, the composition y_i of the gas phase, and the temperature T_i are determined for each increment i.

In the following, the 3 process steps are explained in more detail.

1. Blow down

The blow down step leads to a product stream of concentrated heavy component. During the blow down, the pressure of the adsorption bed is decreased by removing gas. Accordingly, ΔN_i in Equations (S1), (S2), and (S3) is negative and the pressure reduces from p_{ads} to p_{des}. Due to the decreasing pressure, fluid is desorbed, and the concentration y of the heavy component in the gas phase increases. The removed gas ΔN_i is summed up by

$$
N_{\text{product,heavy}} = -\sum_{i=1}^{l} y_i \Delta N_i \tag{S7}
$$

and

$$
N_{\text{product,light}} = -\sum_{i=1}^{l} (1-y_i) \Delta N_i \tag{S8}
$$

to determine how much of the heavy component ($N_{\text{product,heavy}}$) and the light component ($N_{\text{product,light}}$) leaves the adsorption column as the product stream.

For the pressure range below atmospheric pressure, the fluid is removed from the adsorption column by a vacuum pump. The energy W_{vac} necessary for the vacuum pump during the blow down corresponds to the work necessary to drive the process since no other energy inputs are needed in the simplified process model. The energy $W_{i,\text{vac}}$ necessary per increment is calculated by

$$W_{i,\text{vac}} = \begin{cases} \Delta N_i \left(\frac{\kappa_i}{\kappa_i - 1}\right) \frac{1}{\eta} R T_i \left(\left(\frac{p_{\text{atm}}}{p_i}\right)^{\frac{\kappa_i - 1}{\kappa_i}} - 1 \right), & \text{if } p_i < p_{\text{atm}} \\ 0, & \text{if } p_i \geq p_{\text{atm}}. \end{cases} \tag{S9}$$

The energies per increment are summed up to obtain the total energy necessary for the vacuum pump:

$$W_{\text{vac}} = \sum_{i=1}^{l} W_{i,\text{vac}}. \tag{S10}$$

In Equation (S9), η is the isentropic efficiency of the vacuum pump, which is assumed to be constant. The ratio of heat capacities κ_i in Equation (S9) is calculated for each increment i based on the current composition of the gas phase and the ratios of heat capacities of the individual pure components according to

$$c_{p,i}^{\text{mix}} = y_i \frac{\kappa_{\text{heavy}}}{\kappa_{\text{heavy}} - 1} R + (1 - y_i) \frac{\kappa_{\text{light}}}{\kappa_{\text{light}} - 1} R \tag{S11}$$

$$c_{v,i}^{\text{mix}} = c_{p,i}^{\text{mix}} - R \tag{S12}$$

$$\kappa_i = \frac{c_{p,i}^{\text{mix}}}{c_{v,i}^{\text{mix}}}. \tag{S13}$$

For these calculations, the ratios of heat capacities κ_{heavy} and κ_{light} of the pure components are assumed to be constant.

2. Repressurization

During repressurization, feed is added to the adsorber bed. Therefore, ΔN_i in Equa-

tions (S1), (S2), and (S3) is positive and the pressure increases until the adsorption pressure p_{ads} is reached. Due to the increase in pressure, the uptake $q_{\text{ads}}(p, T, y)$ of the adsorbent increases. For simplification, it is assumed that the repressurization step is a well-mixed process, so the desired pressure is reached by sequentially adding small quantities of the feed, which immediately mix with the gas phase inside the adsorber.[4]

The amount of substance ΔN_i entering the adsorption column is summed up to determine the total amount of feed added to the column during repressurization, according to

$$N_{\text{repressurization,heavy}} = \sum_{i=1}^{l} y_{\text{feed}} \Delta N_i \tag{S14}$$

$$N_{\text{repressurization,light}} = \sum_{i=1}^{l} (1 - y_{\text{feed}}) \Delta N_i. \tag{S15}$$

3. Feed

After the adsorption pressure p_{ads} is reached, the composition of the heavy component in the gas phase is lower than in the feed. The outlet of the adsorber bed is opened while feed is continuously added, keeping the pressure in the adsorber bed constant. Thereby, the feed entering the adsorber bed pushes the gas phase with a high concentration of the light component out of the bed. A complete breakthrough is assumed, i.e., the composition of the outflow equals the composition of the gas phase at the end of the repressurization. This assumption avoids calculating the composition of the gas phase within the adsorber column due to mixing with the fluid entering the column. The component balances in the feed step are calculated by

$$\begin{aligned} 0 = {} & q_{\text{ads,heavy}}(p_1, T_1, y_1) \cdot m_{\text{solid}} - q_{\text{ads,heavy}}(p_0, T_0, y_0) \cdot m_{\text{solid}} \\ & + \rho_{\text{gas,heavy}}(p_1, T_1, y_1) \cdot V_{\text{void}} - \rho_{\text{gas,heavy}}(p_0, T_0, y_0) \cdot V_{\text{void}} \\ & + y_{\text{feed}} N_{\text{feed}} - y_1 N_{\text{waste}} \end{aligned} \tag{S16}$$

$$0 = q_{\text{ads,light}}(p_l, T_l, y_l) \cdot m_{\text{solid}} - q_{\text{ads,light}}(p_0, T_0, y_0) \cdot m_{\text{solid}}$$
$$+ \rho_{\text{gas,light}}(p_l, T_l, y_l) \cdot V_{\text{void}} - \rho_{\text{gas,light}}(p_0, T_0, y_0) \cdot V_{\text{void}} \tag{S17}$$
$$+ (1 - y_{\text{feed}})N_{\text{feed}} - (1 - y_l)N_{\text{waste}}$$

with subscript l representing the state after the last increment of the repressurization and subscript 0 the initial state of the blow down.

Since all temperatures and compositions at the beginning and end of the feed step are already known, Equations (S16) and (S17) can be solved analytically to determine how much of the feed stream enters the adsorption column (N_{feed}) and how much substance leaves the adsorption column as waste stream (N_{waste}).

S2 Isotherms of the 3 best-performing materials

In Figures S1–S6, the isotherms calculated by the 1D-DFT model are compared to the isotherms calculated by GCMC simulations[5] for CO2 and N2 and the 3 MOFs performing best in the screening study (BUSQIQ, NAXLII, ELUJEC, see Table 2).

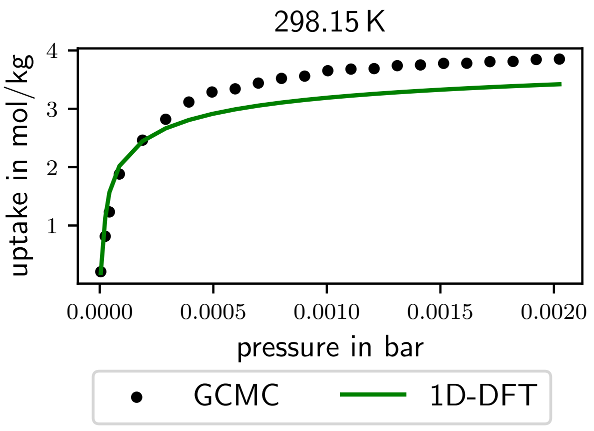

Figure S1: CO_2-isotherm for BUSQIQ.

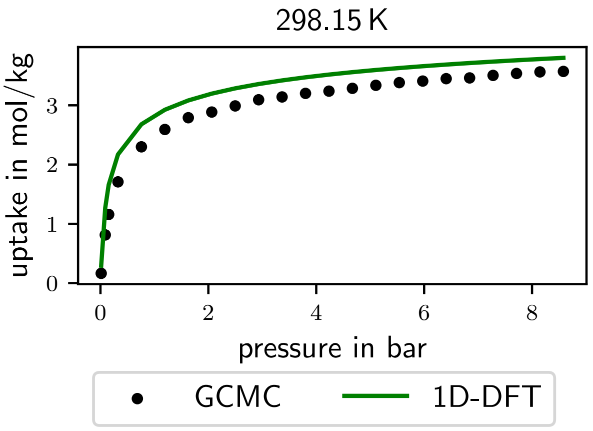

Figure S2: N_2-isotherm for BUSQIQ.

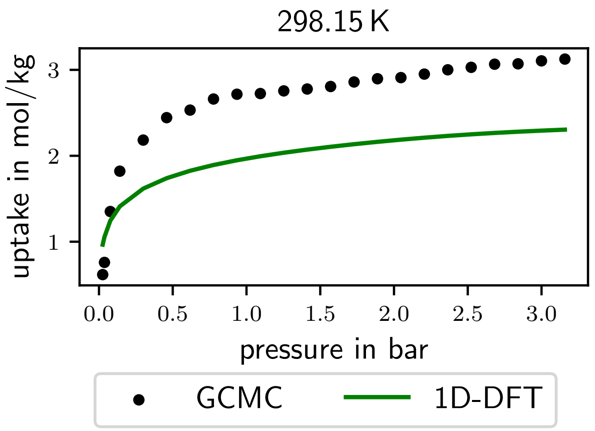

Figure S3: CO_2-isotherm for NAXLII.

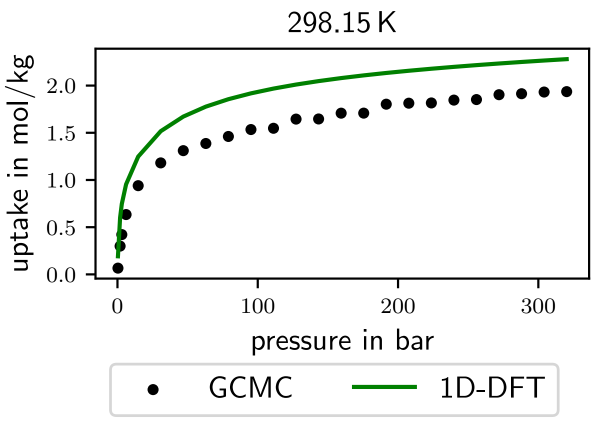

Figure S4: N_2-isotherm for NAXLII.

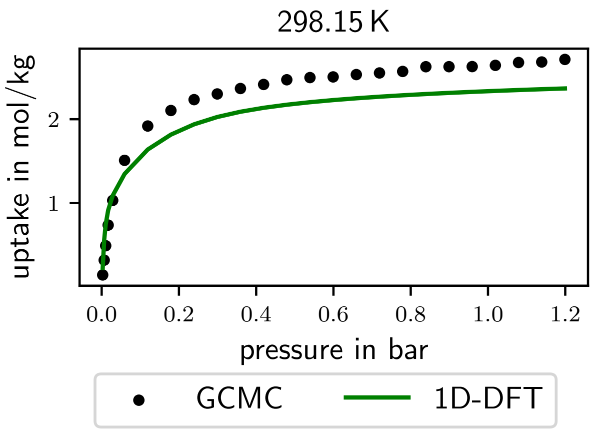

Figure S5: CO_2-isotherm for ELUJEC.

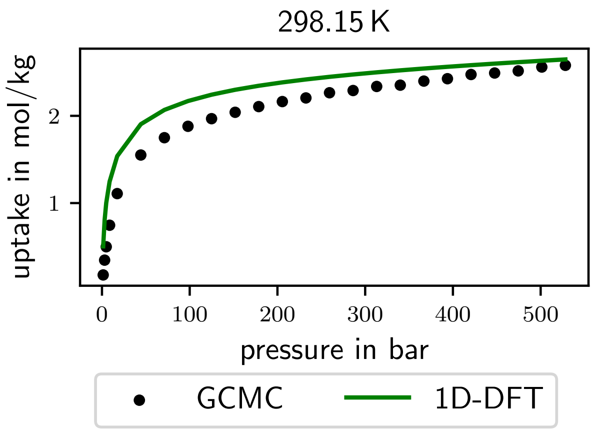

Figure S6: N_2-isotherm for ELUJEC.

In general, the uptake of CO_2 is slightly underestimated by the 1D-DFT model while it is overestimated for N_2. For BUSQIQ and ELUJEC, the 1D-DFT-isotherms are close to the GCMC data while for NAXLII, there are larger deviations. However, the deviations result in underestimation of the actual process performance, since the selectivity shifts towards N_2 due to the systematic deviations of the 1D-DFT model. For this reason, we apply the 1D-DFT model in our screening method despite its deviations. In future work, the 1D-DFT model should be refined to achieve results closer to real-world applications.

References

(1) Maring, B. J.; Webley, P. A. A new simplified pressure/vacuum swing adsorption model for rapid adsorbent screening for CO_2 capture applications. *International Journal of Greenhouse Gas Control* **2013**, *15*, 16–31.

(2) Virtanen, P. et al. SciPy 1.0: fundamental algorithms for scientific computing in Python. *Nature Methods* **2020**, *17*, 261–272.

(3) Gross, J.; Sadowski, G. Perturbed-Chain SAFT: An Equation of State Based on a Perturbation Theory for Chain Molecules. *Industrial & Engineering Chemistry Research* **2001**, *40*, 1244–1260.

(4) Zhang, J.; Webley, P. A. Cycle Development and Design for CO_2 Capture from Flue Gas by Vacuum Swing Adsorption. *Environmental Science & Technology* **2007**, *42*, 563–569.

(5) Moubarak, E.; Moosavi, S. M.; Charalambous, C.; Garcia, S.; Smit, B. A Robust Framework for Generating Adsorption Isotherms to Screen Materials for Carbon Capture. *Industrial & Engineering Chemistry Research* **2023**, *62*, 10252–10265.
